# Senescence protein signatures predict dementia risk with causal implication for TBCA: a two-cohort study

**DOI:** 10.21203/rs.3.rs-9131525/v1

**Published:** 2026-03-26

**Authors:** Anna Prizment, Saeun Park, Zexi Rao, Shuo Wang, Pamela L. Lutsey, Jim Pankow, Shannon M. Sullivan, Weihong Tang, Behnam Sabayan, Timothy M. Hughes, Keenan A Walker, Ruth Dubin, Rajat Deo, Wendy Post, Jerome I Rotter, Alexis C. Wood, Peter Ganz, Weihua Guan, Sanaz Sedaghat

**Affiliations:** University of Minnesota; University of Minnesota; University of Minnesota; University of Minnesota; University of Minnesota; University of Minnesota; University of Minnesota; University of Minnesota; University of Minnesota; Wake Forest University School of Medicine; National Institute on Aging; University of Texas Southwestern Medical Center; University of Pennsylvania; Johns Hopkins University; The Lundquist Institute for Biomedical Innovation, Harbor-UCLA Medical Center; Children’s Nutrition Research Center at Baylor College of Medicine; Zuckerberg San Francisco General Hospital, University of California; University of Minnesota; University of Minnesota

**Keywords:** proteomic score, dementia, senescence, senescent-associated secretory phenotype (SASP), prospective analysis, mendelian randomization

## Abstract

Senescence, a key aging mechanism, is linked to disease via the senescence-associated secretory phenotype (SASP), yet its role in dementia remains unclear. We aimed to identify a minimal circulating SASP protein panel to predict incident dementia and identify causally-associated proteins. Among midlife and late-life participants from the Atherosclerosis Risk in Communities (ARIC) study, we developed three weighted SASP protein scores via multivariable Cox proportional hazards or Lasso Cox regression. Cox proportional hazards regression estimated associations between each standardized score (mean = 0; SD = 1) and incident dementia in an independent ARIC sample and validated in the Multi-Ethnic Study of Atherosclerosis (MESA). Mendelian randomization analyses evaluated causal relationships between SASP proteins and dementia risk. Comparable significant associations were observed across scores in ARIC (per 1 SD: HRs: midlife 1.20–1.33; late-life 1.39–1.50) and MESA (midlife: 1.28–1.38; late-life 1.40–1.62), with stronger associations for late-life scores. Tubulin folding cofactor A protein was the only protein causally associated with incident dementia. SASP scores, even with few proteins, are useful dementia biomarkers, and TBCA is a promising therapeutic target.

## Introduction

About 6.2 million Americans are living with dementia, and this number is expected to reach 13.8 million by 2060 due to the ongoing population aging.^1^ Dementia has a long preclinical phase providing opportunity to intervene before its clinical diagnosis. Thus, there is a need to identify biomarkers that are important for predicting dementia and understanding disease development, and could be targeted by interventions.

Because dementia is an age-related condition, its development is closely related to the accumulation of senescent cells that occur with aging. Senescence is a complex response to stress characterized by activation of the p16INK4a/Rb and p53/p21 pathways, permanent cell cycle arrest, and resistance to cell death.^2–4^ Senescent cells do not divide, but remain metabolically active and have altered patterns of protein expression and secretion compared to non-senescent cells. While senescence evolved as a beneficial mechanism to prevent the division of damaged cells, the age-related accumulation of these cells becomes harmful mainly because they develop a senescence-associated secretory phenotype (SASP). The SASP phenotype is characterized by secreting proteins into circulation, including proinflammatory cytokines, chemokines, proteases, and growth factors.^2^ Importantly, SASP is a selective regulator of protein secretion. While it alters the expression of numerous proteins, it does not affect many others, such as, pro- and anti-inflammatory interleukins (e.g., IL2, IL4, IL10, IL11, or IL12).^2^ The SASP proteins released into blood have been shown to be associated with mortality^5,6^ and morbidity including pulmonary fibrosis, atherosclerosis, osteoarthritis, and type 2 diabetes (reviewed in ^7^).

Emerging evidence from animal studies^8^ show that senescence also contributes to the pathophysiology of neurogenerative diseases including Alzheimer’s disease, the most common form of dementia, while anti-senescence treatments reduce the amyloid β (Aβ) load and tau aggregation and thus delay cognitive decline in mice.^8,9^ In patients with dementia compared to those without, a greater proportion of various brain cells exhibit a senescence phenotype,^10,11^ and the accumulation of senescent cells co-occurs with the buildup of tau-containing neurofibrillary tangles, the hallmark of Alzheimer’s disease.^12^ A recent study of sedentary individuals 70–89 years old found that two SASP proteins, matrix metalloproteinase 7 and myeloperoxidase, were associated with mild cognitive impairment and dementia after two years of follow-up.^13^ However, the generalizability of this finding to other populations is unclear because that study was limited to low-functioning older individuals, a two-year study duration, and a limited number of SASP proteins. Therefore, we used the data from two large population prospective cohorts – the Atherosclerosis Risk in Community (ARIC) study and the Multi-Ethnic Study of Atherosclerosis (MESA) – to comprehensively examine functional SASP proteins that were already validated in previous studies.^14,15^ Unlike most previous studies that examined individual SASP proteins, we investigated three composite scores, each including a distinct panels of SASP proteins associated with dementia. We examined composite scores because the proteins interact through shared pathways and their combined effects are likely better captured by these broader measures. To balance parsimony and performance, we created three scores with the goal to select a score with a limited protein number but a predictive performance comparable to a larger, less parsimonious score. We tested associations of these SASP scores at midlife and late-life with the subsequent risk of dementia in the ARIC independent samples and the MESA study. Finally, we examined individual SASP proteins associated with incident dementia, and applied Mendelian randomization (MR) methods to evaluate potential causal associations with dementia by using genetic instruments from two independent genome-wide association studies.^16^

## Materials and methods

### ARIC study

#### Study population

This study included White and Black men and women – participants of the ongoing ARIC study (RRID: SCR_021769). ARIC study is a large, community-based cohort study launched in 1987, with the goal of identifying the causes and clinical outcomes of atherosclerosis among US adults. The study enrolled 15,792 participants aged 44 to 66 years from four US communities: suburbs of Minneapolis, Minnesota; Washington County, Maryland; Forsyth County, North Carolina; and Jackson, Mississippi.^17,18^ Since baseline (Visit 1, 1987–89), 10 follow-up examinations were completed to date. In this study, we examined plasma proteins measured at Visit 2 (1990–92) and Visit 5 (2011–13). Participants were excluded if they did not have protein data at the visit of interest, were diagnosed with dementia before the corresponding visit, identified their race as other than White or Black, or were Black participants from Minneapolis or Washington County due to extremely small sample sizes. After the exclusions, 11,755 midlife participants from Visit 2 and 4,621 late-life participants from Visit 5 were included in the analysis (**Supplementary figures S1A and S1B**).

The study was approved by each site’s institutional review board, and written informed consent was signed by all participants (or proxies, when required). For this study, we obtained de-identified data through manuscript proposal submissions to the publication committees of both parent cohort studies: ARIC (IRB0031186; University of Minnesota site IRB SITE00000837) and MESA (IRB9805M000340). The study was conducted in accordance with Declaration of Helsinki. Additional ethical review was not required according to the policies of our institute.

#### Measurement of proteomics

Plasma samples from each ARIC study site were collected by a standardized protocol, frozen at − 80°C and shipped on dry ice to the ARIC central laboratory, where they were stored at − 80°C until analysis.^19,20^ About 5,000 proteins, corresponding to 5,284 aptamers, were measured by SOMAscan^™^, a high-throughput, multiplexed DNA-based aptamer assay,^19,21^ in samples from Visit 2 and Visit 5. Of note, the number of aptamers exceeds the number of proteins in our study because multiple aptamers can bind to a single protein. Among the measured aptamers, ARIC excluded those with a Bland-Altman coefficient of variation (CVBA) ≥ 50%, log-scale variance < 0.01, those bound to mouse Fc-antibody, contaminants, non-proteins, or lacked a UniProt ID.^22^ In total, 4,712 proteins (4,955 aptamers) measured at each visit (Visit 2 and Visit 5) were included in this analysis. The CVBA for split samples was 6% at Visit 2 and 7% at Visit 5.^23^ The details about the SOMAscan^™^ assay and data normalization process have been described previously.^19,21^ Protein concentrations were reported in relative fluorescent units (RFU) and transformed into log2 scale to adjust for skewedness; outliers > 5 standard deviations (SD) from the sample mean were winsorized.

#### Dementia assessment

Assessment of incident dementia was described in the previous ARIC studies.^24–26^ Briefly, the 3-instrument cognitive battery (Delayed Word Recall, Word Fluency, and Digit Symbol Substitution) was used Visit 2 and Visit 4 (1996–98), and an expanded battery was administered at Visit 5 as part of the ARIC Neurocognitive Study (ARIC-NCS; 2011–2013). For ARIC participants who were not available for in-person assessment, dementia was also ascertained by participants’ telephone cognitive testing at the time of Visit 5. Additionally, starting in 2012 all participants were invited to take part in twice-yearly telephone interviews which included dementia screening with the Six Item Screener followed up with proxy interviews using the AD8 when appropriate. Additional dementia cases were identified by hospital discharge codes or death certificates.^27^ An expert committee consisting of physicians and neuropsychologists adjudicated dementia status using all available information and pre-determined algorithms based on the National Institute on Aging–Alzheimer’s Association (NIA-AA) work groups and the Diagnostic and Statistical Manual of Mental Disorders, 5th Edition (DSM-5).^25^ The follow-up started from the corresponding visit until dementia diagnosis, death, loss of follow-up or the end of 2019, whatever occurred first.

#### Other covariates

Race (Black and White) and educational attainment (less than high school, high school equivalent, and greater than high school) were self-reported through questionnaires at ARIC Visit 1. Other covariates were measured at both ARIC Visit 2 and Visit 5. Smoking status (current, former, or never users) were self-reported at each visit. Body mass index (BMI) was calculated as weight divided by height squared (kg/m^2^). Diabetes was defined as a self-reported history of physician-diagnosed diabetes, use of diabetes medication within the past two weeks, a fasting blood glucose level ≥ 126 mg/dL, a non-fasting blood glucose level ≥ 200 mg/dL, or a hemoglobin A1C level ≥ 6.5%.^28^ Blood pressure was measured three times by trained technicians with participants seated following a 5-minute rest period; the average of the last two measurements was recorded.^29^ Hypertension was defined as a systolic blood pressure 140 mm Hg or higher, a diastolic blood pressure 90 mm Hg or higher, or the use of antihypertensive medications.^30^ Plasma total cholesterol levels were quantified employing enzymatic methods.^31^ Estimated glomerular filtration rate (eGFR) was calculated using creatinine and cystatin C with the race-free CKD-EPI 2021 equation.^32^

### MESA study

#### Study population

MESA recruited 6,814 men and women aged 45–84 who identified their race/ethnicity as White, Black, Chinese, or Hispanic/Latino and were free of clinical CVD from six locations in the United States (Baltimore City and Baltimore County, Maryland; Chicago, Illinois; Forsyth County, North Carolina; Los Angeles County, California; New York, New York; and St. Paul, Minnesota) in in 2000–2002.^33^. Since the baseline (Exam 1, 2000–02), 6 follow-up examinations were conducted so far: Exam 2 (2002–2004), Exam 3 (2004–2005), Exam 4 (2005–07), Exam 5 (2010–2011), Exam 6 (2016–2018), and Exam 7 (2022–2024). Information about demographic, lifestyle and medical factors was collected at baseline and each follow-up exam. To validate SASP scores created in ARIC, we applied midlife SASP scores to MESA Exam 1 participants (N = 5,829, 44–84 years) and late-life SASP scores to Exam 5 participants (N = 4,065, 53–94 years), because the age ranges of MESA participants at those exams were comparable to those of ARIC midlife (46–69 years) and late-life (67–90 years), respectively. Participants were excluded from the analysis using the exclusion criteria similar to those in the ARIC study or had a dementia diagnosis before the corresponding exam (**Supplementary figures S2A and S2B**).

The institutional review boards at all participating sites approved the study, and all participants provided written informed consent.

#### Measurement of proteomics

In this study, we used plasma samples collected by a standardized protocol at MESA Exam 1 and Exam 5 and then frozen at − 80°C and stored at − 80°C. Proteomic profiling of 6,430 proteins (corresponding to 7,329 aptamers), was performed at SomaLogic using the SOMAscan^™^ v4.1 assay.^23^ This assay includes all aptamers measured by the earlier v4.0 assay used in ARIC. The means and distributions of randomly selected aptamers measured in ARIC and MESA were similar.^26^ The laboratory used standard SOMALogic quality control methods for normalization and calibration. Similar to ARIC, aptamer values were measured in relative fluorescent units (RFU), transformed into log2 scale, were winsorized at 5 SD.^34^

#### Dementia assessment

MESA follow-up interviews collected information on any interim hospital admissions and deaths every 9 months ^35^. Copies of all available death certificates and ICD-10 codes, face sheets and ICD-9 codes from hospital records and some outpatient diagnoses were gathered by MESA staff at each center. Detailed description of the dementia ascertainment and its validity can be found in elsewhere ^35^. Briefly, in MESA, candidate dementia cases were identified using ICD-9 or ICD-10 codes, and a physician blinded to the ICD codes reviewed available medical records for potential dementia cases and ascertained those that were unlikely to be dementia.^36^ Follow-up for each of Exams 1 and 5 participants continued from the corresponding exam date until dementia diagnosis, death, or the end of follow-up in 2018, whichever occurred first.

#### Other covariates

Race/ethnicity (Black, Chinese, Hispanic, and White) and educational attainment were self-reported by questionnaire at Exam 1. Educational attainment was categorized as less than high school, high school equivalent, and greater than high school. Other covariates were collected at both exams. Smoking status (never, former, and current smoker) was self-reported. BMI was calculated as weight divided by height squared (kg/m^2^). Diabetes was defined as fasting glucose > 6.99 mmol/L (126 mg/dL) or use of hypoglycemic medication. Total cholesterol was measured using the cholesterol oxidase method (Roche Diagnostic, Indianapolis, IN).^37^ Resting blood pressure was measured three times and the average of the last two measurements was used. Hypertension was defined as the use of hypertension medication, a diagnosis of hypertension, or SBP ≥ 140 mm Hg or DBP ≥ 90 mm Hg. eGFR was calculated using Chronic Kidney Disease Epidemiology Collaboration equation.^38^

### Statistical analysis

#### Development of SASP scores in the ARIC training set

SASP scores at midlife (Visit 2) and at late-life (Visit 5) in ARIC were created in two-thirds of all participants who were randomly selected into the training set at each ARIC visit (N for Visit 2 = 7,837, N for Visit 5 = 3,081). At each time point, we developed three SASP scores, using different protein panels and training them against dementia, and then constructed all the scores as weighted sums of the proteins.

The first two scores (Score-1 and Score-2) were based on 133 proteins (144 aptamers) of 177 “core” SASP proteins reported in the SASP atlas by Basisty *et al*.,^14^ a comprehensive database of SASP proteins in human plasma.^14,39^ The SASP proteins were included in the atlas because they changed their concentrations under the effect of each of 3 senescence agents (X-ray radiation, RAS, and Atazanivir) in primary human cells by at least 1.5-fold vs. placebo [P-values < 0.05 corrected for false discovery rate (FDR)]. Of 177 SASP proteins in the atlas, 44 proteins were not measured in the ARIC study. For Score-1, the weights were computed by Cox ridge regression model that included all 133 proteins as predictors and dementia until the end of 2019 as the outcome. The model was adjusted for covariates associated with dementia, including chronological age, sex, joint terms for race and study center (Black participants from any center other than Mississippi; White participants from Maryland, North Carolina, or Minnesota), education, BMI, smoking status, diabetes, hypertension, total cholesterol level, and estimated glomerular filtration rate (eGFR). Penalization in the Cox ridge model was applied only to the proteins, not to covariates. The lambda value (0.11 for midlife, 0.20 for late-life) was selected based on a 10-fold cross-validation in the training set.

To create Score-2, we tested each of the 133 proteins (144 aptamers) individually using Cox proportional hazards regression after adjusting for the same covariates as for Score-1, with dementia as the outcome. After applying an FDR-corrected p-value < 0.05, the Cox proportional hazards regression model selected 16 proteins (16 aptamers) at Visit 2 and 6 proteins (6 aptamers) at Visit 5 and their weights.

To create Score-3, we used 7 SASP proteins (9 aptamers) validated in a previous study by Schafer *et al*.^15^ That selected 24 candidate proteins that were consistently upregulated by induced senescence (X-ray radiation) across various human cell types (fibroblasts, preadipocytes, epithelial cells, and myoblasts) and were readily measurable in human plasma. Of 24 SASP proteins. that study selected a sever-protein panel—activin A, CCL3, GDF15, FAS, IL-15, OPN, and TNFR1—as robust predictors of adverse post-surgical outcomes in two patient populations.^15^ We included these 7 SASP proteins together in a multivariable Cox proportional hazards model adjusted for the same covariates as in analyses of two other scores. Thes model identified 2 proteins (3 aptamers) at Visit 2 and 4 proteins (5 aptamers) at Visit 5 as significantly associated with dementia (p-value < 0.2). To determine the weights for the selected proteins, we re-ran a multivariable Cox regression including the same list of covariates but only dementia associated proteins. The protein numbers and selection process for computing each score are presented in Table 1 and the list of proteins in each score, in **Appendix Tables A1–A6**.

### Analysis of SASP scores and dementia risk in the ARIC and MESA studies

We computed three midlife and late-life scores in the independent samples, comprising the remaining one-third of the ARIC participants (test set: 3,918 at Visit 2 and 1,540 at Visit 5) and MESA (5,829 at Exam 1 and 4,065 at Exam 5). Each score was computed as a weighted sum of aptamers: $Score=\sum_{i=1}^{n}\beta_{i}\times {aptamer}_{i}$, where ${aptamer}_{i}$ is the concentration of i^th^ aptamer, and $\beta_{i}$, weight of i^th^ aptamer, as obtained from the training set. We standardized all SASP scores (mean = 0, SD = 1) and employed Cox proportional hazards regression to examine the associations between each score (individually) at each visit and incident dementia. Two models were created: unadjusted and fully adjusted which was adjusted for age, sex, joint race–center terms in ARIC and separate terms for race/ethnicity and study site in MESA, and additionally adjusted for education, BMI, smoking status, diabetes, hypertension, total cholesterol, and eGFR. Of note, the dementia-associated gene APOE ε4 was not included as covariate because its addition did not markedly change the associations between scores and dementia risk.

Furthermore, we evaluated model prediction for incident dementia by computing the concordance index (C-index) from Cox proportional hazards models at midlife and late-life in both studies. We calculated the C-index for the following models: (1) models including each score as the only predictor; (2) models including all covariates (no scores included); and (3) models included all covariates plus each score added.

### Two-sample Mendelian Randomization (MR) analysis

We investigated the potential causal relationship between SASP proteins and dementia risk using Two-sample MR. Summary statistics were obtained from two independent GWAS datasets, one for proteins and another one for dementia (GCST90027158). For the MR analysis we included SASP proteins significantly associated with dementia risk at either midlife or late-life in the ARIC training sets (i.e. those included in Score-2). This resulted in a total of 20 unique proteins (represented by 20 aptamers) available for testing in the MR analysis.

Genetic instrumental variables (IVs), defined as SNPs associated with each protein, were obtained from publicly available GWAS summary data for 35,559 Icelanders in the deCODE Health study.^40^ Because GWAS data was unavailable for three proteins (three aptamers), these were excluded, resulting in 17 SASP proteins (17 aptamers) examined in the MR analysis (**Appendix Table B1**). SNPs used as IVs for each included protein were selected based on the following criteria: minor allele frequency (MAF) > 0.01, genome-wide significance (P < 5 × 10^− 8^), and presence in the dementia GWAS dataset. We applied LD clumping using PLINK with a 1000 Genomes Phase 3 European (EUR) reference panel, pruning SNPs at r^2^ < 0.01 within a 2 Mb window to ensure independent instruments. Summary statistics for SNP–dementia associations were obtained from the deCODE genetics GWAS.^41^ All MR analyses were performed using the TwoSampleMR package (version 0.6.8) in R (version 4.4.2).^42^

The main analysis was conducted using fixed-effects inverse-variance weighting (IVW).^43^ To test the robustness of our findings, we used two additional MR approaches: (1) MR-Egger method^44^ and (2) the weighted median approach.^45^ The two latter methods are more robust to violations of various MR assumptions. For the MR-Egger method, we examined the presence of horizontal pleiotropy, i.e., the influence of SNPs on the outcome independently of the protein level, using the intercept test.^44^ In addition, a leave-one-out analysis was conducted to identify and exclude influential SNPs using the IVW method. MR estimates are reported as beta estimates and odds ratios (OR) and 95% CI, reflecting the effect per one genetically predicted SD increase in protein level. Statistical significance was determined using Bonferroni correction threshold of P < 0.05/ (17*3) = 0.0029, that accounted for 17 tests within each of three MR approaches.

Because tubulin-specific chaperone A (TBCA) was identified as causally associated with dementia risk by all three MR methods, we tested the associations of TBCA with dementia risk at each ARIC and MESA exam. Finally, we examined associations for GDF15 with dementia risk at both life stages for three reasons: a suggestion of causal association with dementia risk by MR IVW method (p = 0.047), GDF15 was the only protein included in all three scores in mid-life and late-life, and it is biological relevant to dementia pathogenesis.^46–51^ Because of strong preliminary GDF15–dementia link, we explored whether GDF15 was driving the association between SASP scores and dementia risk. For this analysis, we excluded GFD15 from Score-2 and examined associations between the reduced Score-2 (without GDF15) and dementia risk at both midlife and late-life in ARIC and MESA. We focused on Score-2 because its protein weights were derived from separate Cox regression models, which allowed us to remove GDF15 without altering the weights of the remaining proteins in the score.

### Additional analyses

We conducted the following additional analyses. First, we explored whether the associations between SASP scores (at two life stages) and dementia are different by sex, race, and APOE ε4 carriership (carrying 1 or 2 ε4 alleles). We stratified by these characteristics and also tested an interaction between SASP score and each characteristic by including their product in ARIC and MESA.

Second, due to the 20-year gap between Visits 2 and 5, many participants were lost to follow-up or died by Visit 5. Thus, we examined the characteristics of those who did and did not participate at Visit 5 exam, and accounted for attrition by applying stabilized inverse probability weighting (IPW) for each participant. To estimate probabilities, we used separate logistic regression models: fully adjusted model adjusted for all-covariates (age, sex, race-center, education, BMI, smoking status, eGFR, hypertension, diabetes, and total cholesterol) and reduced model adjusted for age, sex, race-center. We computed probability of being alive at Visit 5 for all participants in a full [$P({\text{alive}}_{\mathrm{full}})$] and reduced [$P({\text{alive}}_{\text{reduced})}$] models and the probability of attending Visit 5 conditional on being alive at Visit 5 in a full [$P({\text{attendance|alive}}_{\mathrm{full}})$] and reduced [$P({\text{attendance|alive}}_{\mathrm{reduced}})$] models. Stabilized weights were derived as shown in equation shown in Eq. 1: $IPW=\frac{P(\mathrm{alive}{)}_{\mathrm{reduced}}*P(\mathrm{attendance}|\mathrm{alive}{)}_{\mathrm{reduced}}}{P(\mathrm{alive}{)}_{\mathrm{full}}*P(\mathrm{attendance}|\mathrm{alive}{)}_{\mathrm{full}}}$

Extreme values of IPWs were winsorized at the 1st and 99th percentiles.

Finally, because MESA had a wider age range than ARIC, we restricted MESA participants’ age ranges to make them comparable to ARIC’s ages and repeated all analyses.

## Results

### Participants’ characteristics and SASP scores in the ARIC and MESA studies

At midlife (ARIC Visit 2 test set), the mean age of 3,918 participants was 57.0 (SD = 5.7) years, 54.8% were female, and 23.5% identified themselves as Black. By the end of 2019, 758 participants had developed dementia (median follow-up: 23 years). In MESA, participants at Exam 1 (N = 5,829) were on average 62.1 years old (SD = 10.3), 52.0% were female, 39.5% were White, and 26.1% Black, 22.4%, Hispanic, and 12%, Chinese. Among them, 508 developed dementia by the end of 2018 (median follow-up: 16 years). The distribution of participants’ characteristics at midlife and late-life in ARIC and MESA are presented in **Supplementary table S1.**

The SASP Score 1 included all 133 SASP proteins. Score 2 and Score 3 were comprised of distinct proteins with minimal overlap between them or between mid- and late-life; GDF15 was the only protein included in each score at two life stages (**Supplementary figure S3**). There were 20 proteins (20 aptamers) associated with dementia risk at either mid- or late life (i.e., components of Score-2), including TBCA, GDF15, HSPA1A, HSPA1B, and others (**Appendix Table A2, Supplementary table S2**). These proteins are implicated in different biological processes, such as cell proliferation, metabolism, inflammation, and cellular structure maintenance, or they serve as growth factors (**Supplementary table S2)**.

The SASP scores showed moderate to strong correlations with each other in the ARIC test set: r = 0.46–0.81 at midlife and r = 0.40–0.69 in late-life in ARIC (**Supplementary table S3**) and MESA: r = 0.41–0.80 at Exam 1 and r = 0.45–0.74 at Exam 5 (**Supplementary table S4**).

Table 2 presents the distribution of midlife participants’ characteristics across quartiles of midlife Score-1 in ARIC and MESA. In both cohorts at midlife, participants in the higher Score-1 quartile were more likely to be older, non-white, less educated, and tended to be current smokers, have diabetes, hypertension and lower eGFR, which corresponds to worse kidney function. However, only in ARIC, but not in MESA, participants in the higher quartiles of the score tended to be male, and have higher BMI. Similar to midlife, late-life participants in the highest Score-1 quartile tended to be older, have lower education, have hypertension and worse kidney function (**Supplementary table S5)**. Of note, trends in participants’ characteristics across Scores 2 and 3 quartiles at mid- and late-life mirrored those observed for the Score-1 distribution (data not shown).

### Association between SASP scores and dementia incidence in the ARIC and MESA studies

**Supplementary figure S4** presents Kaplan-Meyer curves showing dementia incidence across the quartiles of three midlife and late-life SASP scores from the ARIC cohort (followed for a median of 23 and 6 years, respectively) and the MESA cohort (followed for 16 and 8 years, respectively). In both studies, the dementia incidence was consistently greater for those in the highest compared to lower quartiles of all scores (all *P*-values < 0.0001).

**Figure 1.**
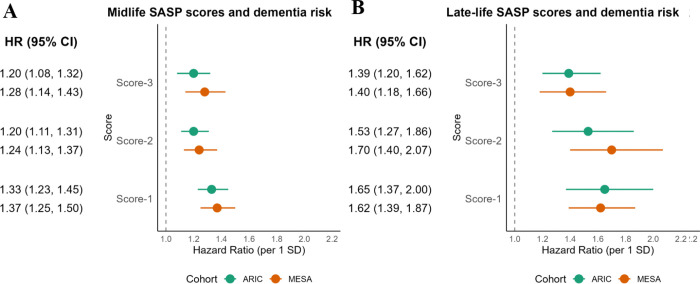
Associations between SASP scores at midlife (ARIC visit 2, N = 3,918; MESA exam 1, N = 5,829) and late-life (ARIC visit 5, N = 1,540; MESA exam 5, N = 4,065) and incident dementia in the ARIC and MESA studies.

The hazard ratios (HR) and 95% confidence intervals (CI) for dementia per 1 SD of each SASP scores modeled as continuous variables are shown in **Supplementary Fig. 1 and Supplementary table S6**. In both unadjusted and fully adjusted models, higher midlife and late-life SASP scores were associated with greater dementia risk in both studies, and all the associations were stronger for late-life compared to midlife scores. At midlife in both studies, the highest HR for dementia was found for Score-1. In a fully adjusted model, one SD higher midlife Score-1 was significantly associated with a 1.33 [95% CI: 1.23, 1.45] and 1.38 [95% CI: 1.26, 1.51] greater risk of dementia in ARIC and MESA, respectively (Fig. 1). HR estimates associated with midlife Score-2 and Score-3 were similar in ARIC and MESA and were slightly lower compared to the HRs for Score-1. In late-life ARIC, the HR was also highest for Score-1 (1.50 [95% CI: 1.28, 1.75], although in late-life MESA the highest HR ([1.70 (95% CI: 1.40, 2.07]) was for Score-2. However, in both midlife and late life, the confidence intervals largely overlapped across three scores.

In parallel to the findings for HRs, predictive performance estimated by C-index was consistently higher in late life compared to midlife across all scores and both studies. The addition of each score to the model with all covariates increased C-index at both times in both studies. In fully adjusted models that included one of the scores and all covariates, C-index was slightly higher for Score-1 (the largest protein panel) in ARIC midlife (0.759), MESA midlife (0.854), and MESA late life (0.819). However, the most parsimonious four-protein Score-3 performed best in ARIC late life (0.771; **Supplementary table S7**).

### Identification of proteins causally associated with dementia risk by the MR analysis

To examine possible causal associations between SASP proteins and dementia risk, we used three MR approaches: IVW, weighted median, and MR Egger, and applied these methods to 17 proteins (17 aptamers) that were associated with dementia risk in our study and had GWAS data available. All three approaches identified only one protein, TBCA (Tubulin-specific chaperone A), as causally inversely associated with dementia risk after Bonferroni correction (P < 0.0024). Of note, the P-values in all three approaches were highly statistically significant (P = 0.008–3.59×10^− 6^) (**Appendix Table B2**). The sensitivity analysis for MR Egger intercept did not detect pleiotropy (P-for intercept = 0.144), and after excluding one TBCA SNP at a time, all the associations remain inverse and significant (**Appendix Table B3**), indicating that the association is robust, with no single SNP disproportionately influencing the overall effect estimate.

In addition, we tested associations between TBCA and dementia risk in ARIC and MESA (Table 3). In agreement with the MR analysis, fully adjusted HRs for dementia associated with TBCA were below 1 at each time point in ARIC and MESA (HR = 0.79–0.92; Table 3), with significant associations observed for all exams except the ARIC late-life exam (Visit 5).

Furthermore, we examined associations for GDF15 with dementia risk at both life stages. GDF15 was consistently associated with higher dementia risk across both studies and timepoints, with stronger associations in late life: fully adjusted HR (95%CI) were 1.41 (1.19, 1.67) in ARIC and 1.43 (1.19, 1.7) in MESA (Table 3). Because of the important role of GDF15, we tested whether excluding GDF15 from Score-2 impacts its association with dementia incidence. For both midlife and late-life score in both studies, all positive associations with dementia remained significant, experiencing only slight attenuation, (**Supplementary table S8**).

### Additional analyses

A sex-stratified analyses in the ARIC study, showed stronger associations for midlife scores among women, but stronger association for late-life scores among men. This pattern, however, was not replicated in the MESA study (**Supplementary table S9**). A race-stratified analyses showed no effect modification in any study, although MESA late-life HR appeared to be somewhat stronger in Hispanic participants for all scores and Chinese participants for Scores 1 and 3 (**Supplementary table S10**). There was no effect modification by Apoe ε4 status for any score, timepoint or study population (**Supplementary table S11)**. Another exploratory analysis found that ARIC participants who missed Visit 5 were older at baseline (Visit 2) and had a less favorable cardiovascular risk profile compared to those who attended the visit (**Supplementary table S12**). After accounting for potential attrition over the 20-year ARIC follow-up, all initial associations remained significant, with some slightly attenuated and others strengthened (**Supplementary table S13)**. Finally, after restricting MESA participants to the age range in the ARIC cohort, the associations for all scores remained significant and estimates became stronger in most of the analyses (**Supplementary table S14**).

## Discussion

In this study, we created and validated SASP protein scores associated with incident dementia at mid- and late-life. Greater values for all SASP scores at both life stages were associated with greater dementia risk in both ARIC and MESA studies. Across all scores and studies, hazard ratios (HRs) for dementia were comparable at each timepoint, with slightly higher HRs for the first score in all analyses, except for the late-life MESA score. The findings of the comparable associations across different SASP protein panels despite their limited overlap, may arise from two factors: the moderate-to-strong correlations between SASP scores likely reflect the shared senescence-related or other underlying mechanisms, and, the complex, multifactorial nature of dementia, where various proteins contribute to the same or interconnected biological processes or pathways.^52–54^

In our study, the HR estimates for dementia were consistently lower for midlife scores than for late-life scores, likely because the late-life protein assessment occurred closer to dementia diagnosis. The finding that associations exist with midlife scores is critical because it suggests that the alterations in dementia-associated SASP proteins begin in middle age and impact the long-term development of dementia diagnosed years later.

Epidemiological research of senescence and dementia is just starting to emerge. One prior study examined 27 SASP proteins in 1,635 low-functioning old adults (aged 70–89) and found that two SASP proteins, myeloperoxidase and matrix metalloproteinase 7, were associated with a composite outcome of mild cognitive impairment or dementia after two years of follow-up.^13^ Another, cross-sectional, study of 326 participants reported a correlation between higher SASP index and worse global cognitive performance, executive dysfunction, slower processing speed, and episodic memory deficits but the temporal relation could not be established due a single-point study design.^55^ To the best of our knowledge, our study is the first to prospectively examine associations between SASP proteins and the incidence of dementia in general population followed for a long period of time. Our findings of the associations between circulating SASP scores and dementia risk are consistent with previous evidence. Prior studies have shown that greater SASP levels were correlated with increased tau levels, inflammation, and cognitive impairment in patients with Alzheimer’s disease, as well as reduced neurogenesis and worse cognitive function in aging mice (reviewed in ^56^). Studies have also shown that senescent cells may drive neuroinflammation, disrupt protein synthesis, and produce misfolded proteins, which cause neuronal damage, the accumulation of amyloid plaques and tau tangles, and contribute to the progression of Alzheimer’s disease.^57^ This biological mechanism aligns with our findings that SASP proteins strongly associated with dementia risk were involved in immune response, inflammation, and cellular and matrix structure.

Of all the SASP proteins associated with dementia risk in our study (N = 17), only one protein, tubulin folding cofactor A (TBCA), was causally and inversely associated with dementia risk as was shown by three mendelian randomization (MR) approaches. A genetically-predicted causal inverse association between TBCA and dementia incidence mirrored the findings of two previous MR studies that reported a protective role of TBCA in both late^58,59^ and early onset of dementia.^59^ A likely explanation for these findings is the functional role of TBCA in the central nervous system, particularly its involvement in neuronal regulation.^60^ TBCA is involved in folding of beta-tubulin and the assembly of the neuronal cytoskeleton, which supports neuronal development, migration, differentiation, and the maintenance of neuronal connections.^61,62^ Conversely, TBCA’s deficiency and abnormalities result in tubulin loss and subsequent tau accumulation and dementia-related brain abnormalities.^63,64^ Because TBCA is an easily measurable SASP protein, whose concentration changes in response to the anti-aging medication, it holds promise as a cost- and time-efficient surrogate marker for biological aging and dementia in clinical trials. Due to the potential role of TBCA in delaying the onset of dementia, it should be explored as a potential therapeutic target for senomorphic medications, which are designed to modulate SASP protein levels.^65^

Another critical SASP protein associated with dementia risk in our study is GDF15, the only protein incorporated in all SASP scores both at midlife and at late-life. GDF15 is a systemic biomarker of oxidative stress, energy metabolism, inflammation, and cellular aging.^46^ Many epidemiological studies showed that circulating GDF15 is associated with different age-related diseases, such as obesity, diabetes, CVD, cancer and dementia,^46–51^ with an especially strong association with vascular dementia.^51^ In parallel with human studies, laboratory studies found that GDF15 is associated with proliferation of neural stem cells and neuronal differentiation and is involved in hippocampal neurogenesis. This suggests that GDF-15 may contribute to dementia development.^66,67^ In spite of strong evidence linking GFD15 to dementia, we did not find a genetically predicted association between GFD15 and dementia risk: only one of three MR approaches (IVW) showed a marginally significant P-value. Our finding of the lack of causal GFD15-dementia association is supported by a large MR study by Zonneveld *et al*.^68^ However, our null finding is in contrast with an earlier, smaller MR study by Wu *et al*. (2021), who reported a potential causal association with Alzheimer’s disease.^69^ Although the reasons for the discrepancies are unclear, the inconsistent results may be due to the differences in GDF15 assays, study populations, and larger datasets in our and Zonneveld’s studies compared to Wu’s. Given the robust associations between circulating GDF15 and dementia risk in our and other epidemiological studies and probable biological mechanisms, future clinical studies are needed to clarify GDF15’s role in dementia.

The strengths of our study include a large panel of SASP proteins measured by the SOMAscan^™^ assay in two population cohort studies – ARIC study of White and Black participants and the MESA study that additionally included Chinese and Hispanic individuals. Other strengths include the development and testing of multiple SASP scores at midlife and late-life, dementia cases prospectively ascertained over a long period of time (during 1993–2020 in ARIC and 2001–2020 in MESA) and detailed information about multiple confounders. A limitation of our study is that the plasma samples were stored for a long period of time; however, these samples have never been thawed and the CV for the oldest samples from ARIC Visit 2 were less than 10% based on blind duplicates, which was comparable to samples collected 20 years after. Another limitation is that the cellular and tissue source of plasma proteins in our study is not known and some proteins could originate from non-senescent cells. This limitation was mitigated by only including previously validated SASP proteins in our scores.^14,15^ Further support for examining circulating SASP proteins comes from animal studies showing a correlation between senescence burden and circulating SASP proteins.^70,71^

In conclusion, replication of three SASP scores for dementia risk in both the ARIC and MESA prospective population-based studies confirms their potential as biomarkers for dementia risk. Although the multivariable-adjusted risk estimates for dementia varied slightly across the scores with lower estimates for the smallest protein panel across both studies and timepoints, the overlap of confidence intervals suggests comparable associations. These findings indicate that a SASP score, even one based on a limited number of proteins, could help clinical risk stratification and therapeutic decision-making, especially when integrated with other dementia-related markers. Future studies should therefore focus on creating SASP scores with a limited number of proteins that may be easily measured by routine clinical analyzers and thus be more readily implementable in practice.

## Supplementary Material

Supplementary Files

This is a list of supplementary files associated with this preprint. Click to download.

• Supplementalfiguresandtables.pdf

• Appendix1.xlsx

• Appendix2.xlsx

• Table2.docx

## Figures and Tables

**Table 1:** Development of SASP scores at midlife (ARIC Visit 2; N = 7,837) and late-life (ARIC Visit 5; N = 3,081) training sets.

Score	Score-1		Score-2		Score-3	
No. of SASP proteins (aptamers)	midlife: 133 (144)	late-life: 133 (144)	midlife: 16 (16)	late-life: 6 (6)	midlife: 2 (3)	late-life: 4 (5)
Source of SASP proteins	133 SASP proteins from the SASP atlas by Basisty [2020]				7 SASP proteins validated by Schafer [2020]	
Selection of SASP proteins and weights estimation	Computed weights using Cox Ridge regression by training all 133 proteins against dementia in a multivariable-adjusted model.^1^		Identified dementia-associated proteins and computed their weights using Cox regression by including each protein individually in a multivariable-adjusted model (FDR adjusted P-value< 0.05).		Identified dementia-associated proteins from a multivariable-adjusted Cox model and estimated their weights by including all proteins in the same model (p < 0.2). Weights were computed by rerunning dementia-associated proteins.	

Abbreviations: ARIC - Atherosclerosis Risk in Communities; FDR - False Discovery Rate; SASP - Senescence-Associated Secretory Phenotype.

1 The model was adjusted for chronological age, sex, joint terms for race and study center, education, BMI, smoking status, diabetes, hypertension, total cholesterol level, and eGFR at corresponding visit.

**Table 3:** Associations of TBCA and GDF15 at midlife (ARIC Visit 2: 1990-1992; MESA Exam 1: 2000-2002) andlate-life participants (ARIC Visit 5: 2011-2013; MESA Exam 5: 2010-2011) with dementia risk^1^.

No. of dementia cases	ARIC Visit 2 (N = 3,918)	MESA Exam 1 (N = 5,829)
	758	508
Total person-years	81,572		83,386	
Protein^2^	HR (95% CI)^3^	P-value	HR (95% CI)^3^	P-value
TBCA	0.79 (0.73, 0.85)	2.05e-10	0.87 (0.80, 0.95)	2.63e-03
GDF15	1.19 (1.08, 1.32)	7.22e- 4	1.31 (1.16, 1.47)	9.57e-06
	ARIC Visit 5 (N = 1,540)		MESA Exam 5 (N = 4,065)	
No. of dementia cases	241		215	
Total person-years	9,365		28,584	
Protein^2^	HR (95% CI)^3^	P-value	HR (95% CI)^3^	P-value
TBCA	0.92 (0.80, 1.07)	0.27	0.79 (0.69, 0.91)	1.37e-03
GDF15	1.41 (1.19, 1.67)	7.26e- 5	1.43 (1.19, 1.71)	1.33e-04

Abbreviations: ARIC - Atherosclerosis Risk in Communities; MESA - Multi-Ethnic Study of Atherosclerosis; TBCA - total brain cerebral atrophy; GDF15 - growth differentiation factor 15; CI - confidence interval; HR - hazard ratio.

1 In ARIC, follow-up was through the end of 2019 (visit 7); in MESA, follow-up was through the end of 2018.

2 All proteins were standardized to the mean (mean = 0, SD = 1).

3 All models were adjusted for chronological age, sex, joint race-center terms in ARIC (Blackparticipants from Mississippi; Black participants from other centers; White participants fromMaryland, North Carolina, and Minnesota), and separate terms for race and study site in MESA,education, BMI, smoking status, diabetes, total cholesterol level, hypertension, and eGFR at thecorresponding visit.

## Data Availability

Data were obtained via an approved proposals by the ARIC (https://aric.cscc.unc.edu/aric9/) and MESA (https://mesa-nhlbi.org) Study Committees. Data requests should be submitted to each committee and will be promptly reviewed for confidentiality or intellectual property restrictions before any data are released. Individual level patient or protein data may further be restricted by consent, confidentiality or privacy laws/considerations. These policies apply to both clinical and proteomic data in both studies. ARIC and MESA data can also be accessed via BioLINCC (ARIC-BioLINCC and MESA-BioLINCC) free of charge and without the need for the ARIC Study approval. There may be some differences in the data available from BioLINCC, such as the removal of extreme values and the omission of restricted data
